# Efficacy and Safety of Prophylactic Mesh Reinforcement for the Prevention of Incisional Hernia: An Umbrella Review of Meta-Analyses

**DOI:** 10.3389/jaws.2026.15631

**Published:** 2026-02-23

**Authors:** Edgard Efren Lozada Hernandez, Luis Alberto Fernández-Vázquez-Mellado, Ricardo Reynoso Gonzalez, Luis A. Martin-del-Campo, Hector Ali Valenzuela Alpuche, H. Alejandro Rodríguez, Enrique Ricardo Jean Silver, Cesar Felipe Ploneda Valencia, Marian Serna Murga, Gloria Valeria Martinez Gonzalez

**Affiliations:** 1 Hospital Regional de Alta Especialidad del Bajío, Guanajuato, Mexico; 2 Hospital Angeles Queretaro, Santiago de Querétaro, Mexico; 3 Hernia care, Ciudad de Mexico, Mexico; 4 Hospital Angeles del Carmen, Guadalajara, Mexico; 5 Hospital Angeles Andares, Guadalajara, Mexico; 6 Tecnologico de Monterrey, Monterrey, Mexico; 7 Asociacion Medica del Centro Medico ABC, Mexico City, Mexico; 8 Universidad Anahuac Mexico - Campus Sur, Mexico City, Mexico

**Keywords:** abdominal wound dehiscence, incisional hernia prevention, midline laparotomy, prophylactic mesh, umbrella review

## Abstract

**Introduction:**

Incisional hernia (IH) is a frequent and expensive complication of laparotomy, occurring in up to 50% of high-risk patients. Although prophylactic mesh placement has been proposed as an effective preventive strategy of IH, its adoption remains limited due to concerns about mesh-related complications and the heterogeneity and variable quality of the available evidence. This umbrella meta-analysis aimed to synthesize the existing evidence to evaluate the efficacy and safety of prophylactic mesh reinforcement for IH prevention.

**Methods:**

A systematic search of multiple databases was performed until June 2025 to identify meta-analyses comparing the use of prophylactic meshes versus primary closure in adults undergoing laparotomy. Methodological quality was assessed with the AMSTAR-2, and the data were reanalyzed with random or fixed effects models. Heterogeneity (I^2^), study overlap (CCA), publication bias, and robustness of the results were evaluated.

**Results:**

Twenty-one meta-analyses were included. Prophylactic mesh reinforcement was associated with a significant reduction in the odds of incisional hernia (OR = 0.29; 95% CI: 0.22–0.38); this effect was consistent across different surgical settings. Mesh use was also associated with an increased risk of surgical site infection (OR = 1.17; 95% CI: 1.04–1.30) and seroma formation (OR = 2.31; 95% CI: 1.99–2.67). No significant differences were observed in abdominal wound dehiscence or hematoma. Overall, the evidence demonstrated a large and consistent effect, although substantial heterogeneity and signs of publication bias were present.

**Conclusion:**

Prophylactic mesh reinforcement is associated with a reduced likelihood of incisional hernia but an increased risk of seroma and surgical site infection. Its use should be considered selectively in high-risk patients, balancing potential benefits against known complications. Further studies are needed to optimize patient selection and evaluate strategies to reduce mesh-related adverse outcomes, as well as to assess cost-effectiveness and quality-of-life outcomes.

## Introduction

Incisional hernia (IH) is the most significant complication of laparotomy, with an incidence of 10%–30% in elective surgeries and up to 50% in high-risk patients [1, 2]. The development of this condition entails high healthcare costs and the need for frequent reoperations [3]. The significant clinical and economic burden associated with this complication calls for the use of preventive strategies. Currently, these efforts are focused on optimizing abdominal wall closure techniques and on the selective use of prophylactic mesh, for which multiple clinical trials have already been published [1].

The meshes applied in the abdominal wall (both for the prophylactic and direct management of IH) mainly function by increasing the tensile strength of the fascia. This resistance must be sufficient to counteract the forces that are exerted on the abdominal wall during activities daily life. Generally, most commercially available meshes exceed the tensile strength required to withstand these physiological forces [4]. However, mechanical failure has been reported to be associated not only with the properties of the material but also with patient risk factors, such as increased intra-abdominal pressure (due, e.g., to obesity), weakness of the connective tissue (as occurs when an aneurysm is present in the abdominal aorta) or the presence of surgical site infections. These conditions can compromise mesh performance and are related to failure to prevent hernia development or recurrence [5].

Although prophylactic mesh placement has been shown in several studies to significantly reduce the incidence of IH compared with primary closure [6], most surgeons do not use it for this purpose. Additionally, only 45% of surgeons claim to be familiar with the literature and use the meshes for this purpose [7]. The main reason for not using these meshes is a fear of infections or other associated complications, reflecting uncertainty regarding morbidity and appropriate patient selection, despite several meta-analyses reporting no overall increase in major complications [7, 8].

Although global evidence indicates a benefit from the use of prophylactic mesh, guidelines such as those of the European Hernia Society (EHS) recommend caution prior to its application, given that the quality of the evidence is low and the strength of the recommendations is weak. This caution is not only related to the heterogeneity and variable quality of the available evidence, but also to concerns regarding mesh-related morbidity and the lack of clear patient-level risk stratification to identify those most likely to benefit. Specifically, the indications for prophylactic mesh use have been evaluated in very different clinical settings (emergency surgery, obese patients, and aortic aneurysms, among others), and the mesh positions, materials and placement techniques differ among the studies. This variability has prevented the implementation of universal and widely applicable recommendations [9, 10].

Although multiple meta-analyses have addressed specific populations and scenarios, no integrative synthesis has systematically compared and ranked the evidence on the use of prophylactic meshes in laparotomy. An umbrella meta-analysis represents the most appropriate methodology for addressing this gap in the field, as it allows the results of different meta-analyses to be reanalyzed uniformly while evaluating the robustness of the findings, quantifying study overlap and classifying the strength of the evidence [11]. Therefore, the objective of this study was to perform an umbrella meta-analysis of the meta-analyses that have been published on this topic, in order to evaluate their effectiveness and safety in the prevention of IH. The findings of this review could provide a solid basis for the development of clinical practice guidelines and international consensus, as they would represent one of the highest levels of evidence in the methodological hierarchy.

## Methods

### Protocol and Registration

An umbrella meta-analysis was performed and reported in accordance with corresponding guidelines, including the Preferred Reporting Items for Overviews of Reviews (PRIOR) [12] and Preferred Reporting Items for Systematic Reviews and Meta-Analyses (PRISMA) guidelines [13]. This study was registered with the hospital research and research ethics committees of the hospital (registration numbers: CEI/HRAEB/004/2025 and CEI-002-2025) and in the International Registry for Prospective Systematic Evaluation (PROSPERO, registration number: CRD420251125560) [14].

### Search Strategy

A systematic search of the English-language literature was performed in the PubMed, The Cochrane Library, SCOPUS, ScienceDirect, and Google Scholar databases from inception to June 30, 2025.

A combination of controlled terms (MeSH) and free terms was used to maximize the sensitivity of the search strategy. Similarly, the bibliographic references of the included studies were manually reviewed to identify relevant articles that may have been missed in the automated search. The search terms included (“Incisional Hernia” [tiab] OR “Abdominal Wall Hernia” [tiab]) AND (“Mesh” [tiab] OR “Prophylactic Mesh” [tiab] OR “Mesh Reinforcement” [tiab]) AND (“Laparotomy” [Mesh] OR “Abdominal Surgery” [tiab] OR “Midline Laparotomy” [tiab]) AND (“Systematic Review” [Publication Type] OR “Meta-Analysis” [Publication Type] OR “systematic review” [tiab] OR “meta-analysis” [tiab]) (Supplementary Table A).

### Inclusion Criteria

This umbrella meta-analysis included only systematic reviews with meta-analyses that provided pooled estimates of at least one of the main outcomes. Studies that met the following PICO criteria were eligible: P (population), adult patients undergoing laparotomy in different surgical settings (including elective, emergency, high risk, abdominal aortic aneurysm, and bariatric surgery, among others); I (intervention), prophylactic mesh placement during laparotomy closure, regardless of the placement plane (onlay, sublay, preperitoneal, or retromuscular) or the type of material used (permanent synthetic, absorbable, or biological); C (comparator), conventional primary closure without the use of a prophylactic mesh; and O (outcomes), including primary outcomes (incidence of IH) and secondary outcomes (mesh-related complications such as SSIs, seroma, chronic pain, and reoperation). Only meta-analyses that presented the full text available in English were included.

### Exclusion Criteria

Narrative reviews, scoping reviews or systematic reviews without quantitative meta-analyses; original articles, editorials, letters to the editor or experimental studies in animals; studies that were exclusively performed in the pediatric population, adolescents or pregnant women; and meta-analyses that exclusively compared different mesh placement techniques without including a primary closure group.

### Selection of Studies and Quality Assessment

Two investigators independently performed the study selection and data extraction processes. The titles and abstracts were subsequently reviewed for preliminary study selection. Finally, a complete reading of the selected texts was performed to determine their eligibility according to the previously defined inclusion and exclusion criteria. Disagreements were resolved via discussion between the investigators; if disagreements persisted, a third evaluator was consulted to make the final decision. The information extracted from each study included the name of the first author, year of publication, country of origin, type of surgery, sample size, follow-up, number of IH patients and reported complications.

### Assessment of Methodological Quality

The methodological quality of the included systematic reviews was assessed independently by two researchers with the AMSTAR (A MeaSurement Tool to Assess systematic Reviews) 2 tool [15]. This tool is specifically designed to evaluate systematic reviews through 16 items encompassing critical domains. The tool classifies the confidence in the results of each review as follows: high confidence, which indicates no critical weaknesses; moderate confidence, which indicates one critical weakness; low confidence, which indicates more than one critical weakness; and critically low confidence, which indicates a relatively large number of critical weaknesses. In this umbrella meta-analysis, only systematic reviews classified as moderate to high quality according to the AMSTAR 2 were included.

### Statistical Analysis

The data were extracted from the included meta-analyses and reanalyzed to guarantee a uniform methodology in the calculation of the risk estimates. Statistical analysis was performed in R Studio (version 1.4.1106) with the R programming language (version 4.3.0) and the metaumbrella package, as well as functions from the meta and metafor packages. The measures of association (OR) were log transformed and subsequently reported in their original metrics via the corresponding exponential.

Statistical heterogeneity was assessed by using the I^2^ statistic and the Cochran Q test. Heterogeneity was considered to be low when I^2^ was <50% and high when I^2^ was >50%. In addition, τ^2^ was calculated as an estimator of the variance between studies. Depending on the degree of heterogeneity, a fixed-effects or random-effects model (DerSimonian–Laird method) was applied. Similarly, 95% prediction intervals (95% PIs) were calculated for each association to estimate the expected variability of the effects in future studies. Forest plots were constructed to visualize the main associations in terms of outcomes and clinical contexts.

The robustness of the findings was evaluated via sensitivity analysis by sequentially eliminating each included meta-analysis (via the leave-one-out method) and recalculating the global estimate. A result was considered to be robust when the exclusion of an individual study did not substantially modify the magnitude, direction or statistical significance of the effect. Publication bias was assessed by using Egger’s regression (considered to be significant if *p* < 0.10 in analyses with ≥10 studies).

The overlap of the primary studies between meta-analyses was quantified with the corrected covered area (CCA) index, which was calculated with the following formula:

$$CCA=\frac{N-r}{\left(r*c\right)-r}$$

where N is the total number of primary study citations (counting duplicates), r is the number of unique primary studies, and c is the number of included meta-analyses. The interpretation was categorized as follows: 0%–5% (slight overlap), 6%–10% (moderate overlap), 11%–15% (high overlap), and >15% (very high overlap) [16]. Finally, the strength of the evidence was classified according to the criteria proposed by Ioannidis and Fusar-Poli, with the evidence being stratified as strong, highly suggestive, suggestive, weak or not significant [17].

## Results

### Study Selection

**FIGURE 1:**
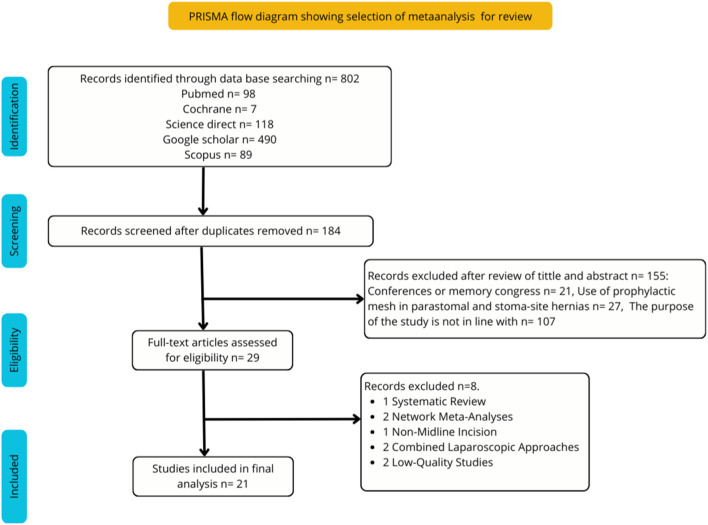
PRISMA flow diagram showing selection of meta-analysis for review.

After searching the different databases, a total of 802 records related to the topic were identified. Following the removal of 618 duplicate articles, 184 records remained for screening. After title and abstract review, 155 articles were excluded for not meeting the inclusion criteria. Consequently, 29 full-text articles were assessed for eligibility. Of these, 8 meta-analyses were excluded after full-text evaluation due to methodological or conceptual reasons, including low methodological quality according to AMSTAR-2 [2], network meta-analysis design [2], non-midline incisions [1], or combined laparoscopic approaches [2]. Finally, 21 meta-analyses were included in the umbrella meta-analysis [1–3, 5, 18–34]. The detailed study selection process and reasons for exclusion are presented in Figure 1.

### Study Characteristics

The included studies were published by researchers in multiple countries, including the United Kingdom [6], the United States [5], the Netherlands [3], Brazil [2], Italy [2], and Germany, Canada and Egypt (one study each). In terms of the type of surgery, 10 studies involved general surgery (elective and emergency), 3 studies involved elective surgery alone, 3 studies involved emergency surgery alone, 3 studies involved abdominal aortic aneurysm surgeries, and 2 studies involved bariatric surgery. Table 1 describes the characteristics of the studies. All studies with adequate methodological quality were evaluated via the AMSTAR 2 scale (Supplementary Table B).

**TABLE 1:** Characteristics of the included studies.

Author(Year)	Country	Patients included	Studies included	Type of patient	AMSTAR 2 rating	Follow up mean
Abbas et al. [18]	Egypt	2,233	15	Elective	MOD	39
Aiolfi et al. [19]	Italy	2,332	14	General	HIGH	32
Olavarría et al. [20]	USA	1,768	15	General	MOD-HIGH	24
Albendary et al. [21]	UK	817	6	Emergency	HIGH	11
Jairam et al. [5]	USA	1,815	12	General	HIGH	32
Hew et al. [22]	UK	487	5	AAA	HIGH	43
Bhangu et al. [23]	UK	588	7	General	MOD	28
Pianka et al. [24]	Germany	1,136	5	Bariatric	HIGH	32
Borab et al. [25]	USA	2,114	14	Elective	HIGH	28
Burns et al. [26]	UK	288	2	Emergency	MOD-HIGH	16
Chou et al. [3]	USA	2,900	19	General	HIGH	29
Dasari et al. [2]	USA	1,095	7	Bariatric	HIGH	17
Frassini et al. [27]	Italy	2,659	16	General	HIGH	33
Indrakusuma [1]	Netherlands	388	4	AAA	HIGH	30
Nachiappan et al. [28]	UK	1,219	9	Elective	HIGH	32
Hassan et al. [30]	Canada	916	7	General	MOD-HIGH	27
Timmermans et al. [31]	Netherlands	346	5	General	HIGH	34
Marcolin et al. [32]	Brazil	464	4	Emergency	HIGH	15
Valerio-Alves et al. [33]	Brazil	2,108	15	General	HIGH	24
Payne et al. [29]	UK	727	8	General	MOD-HIGH	33
van der Berg et al. [34]	Netherlands	493	5	AAA	MOD-HIGH	24

Type of patient. General: refers to meta-analyses that included both elective and emergency patients. AMSTAR 2 Rating: MOD: Moderate Quality, HIGH: High Quality, MOD-HIGH: moderate-high quality. AAA: Abdominal Aortic Aneurysm

### Efficacy: IH and AWD

#### Incisional Hernia

**FIGURE 2:**
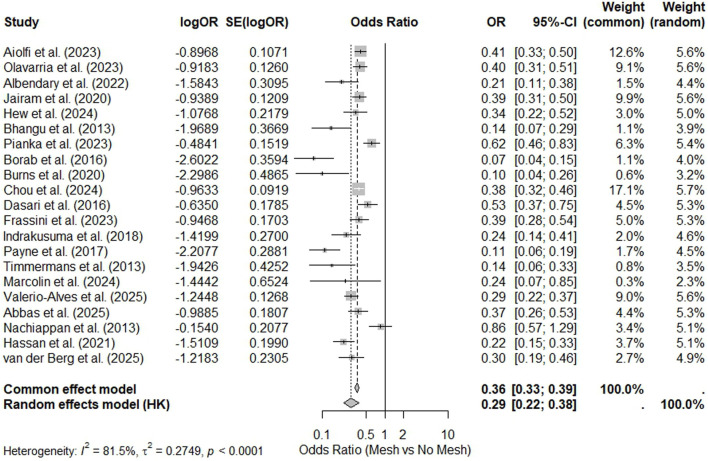
Forest plot, umbrella meta-analysis for Incisional Hernia.

The umbrella meta-analysis revealed that the use of a prophylactic mesh reduces the risk of IH compared with primary closure. The global estimate yielded an OR of 0.29 (95% CI: 0.22–0.38), which corresponds to a relative risk reduction of approximately 71% (Figure 2). The sensitivity analysis by type of surgery revealed that the effect was maintained in elective and emergency procedures as well as in specific populations (including bariatric and AAA surgery patients). Comparisons between meta-analyses with ≥10 studies and those with <10 studies did not reveal changes in the direction or magnitude of the effect (Table 2).

**TABLE 2:** Sensitivity analysis by surgery type and >10 articles.

Surgery type	Studies (n)	I^2^ (%)	p (Heterogeneity)	Model	OR	95% CI
1. By surgery type						
General	10	77.3	<0.0001	Random effects	0.29	0.22–0.38
Emergency	3	0	<0.41	Fixed effects	0.18	0.11–0.28
Elective	3	91.2	<0.001	Random effects	0.58	0.46–0.73
AAA	3	0	0.61	Fixed effects	0.3	0.23–0.39
Bariatric	2	0	0.51	Fixed effects	0.58	0.46–0.73
2. By > or <10 articles						
>10	8	72.5	<0.0006	Random effects	0.33	0.23–0.49
<10	13	85.4	<0.0001	Random effects	0.27	0.18–0.40

The overall heterogeneity was high (I^2^ = 81.5%). The funnel plot showed asymmetry, and the results of the Egger test were significant (p = 0.008), thus suggesting publication bias (Supplementary Figure A). The leave-one-out analysis confirmed that the results were stable; specifically, after each meta-analysis was sequentially excluded, the OR remained between 0.30 and 0.42, thus excluding the greatest influence of an individual study (Supplementary Figure B).

Overlap analysis of the primary studies included in the meta-analyses revealed a CCA index of 0.18, indicating a moderate degree of overlap [35]. Thirty-eight unique studies were identified as the basis of the available evidence.

Global analysis of excess significance revealed that the number of studies with observed positive results (n = 20) was greater than expected according to the mean statistical power (16.9). This corresponds to an excess significance of 14.9% (p = 0.018), which indicates the possible presence of publication bias or overreporting. In the subgroup analyses (including AAA, bariatric, elective, emergency and general surgical subgroups), no excess of significance was identified (p > 0.05).

The 95% prediction interval was calculated, which estimates the expected range for the effect size of an individual future study from the same study population. The obtained interval (0.22–0.37) indicates that, with 95% confidence, the *odds ratio* of a new study would be observed within this range. Since the entire interval is well below 1.0, the protective effect of the prophylactic mesh was considered to be robust and consistently beneficial for preventing IH in various clinical settings.

Despite the consistent direction of effect observed across the included meta-analyses, the overall certainty of the evidence is downgraded by substantial heterogeneity, signals of publication bias, and the presence of excess significance. These methodological features reflect limitations inherent to the available meta-analytical evidence and should be considered when interpreting the magnitude and generalizability of the pooled estimates at the umbrella review level.

#### Classification of the Evidence

According to the Ioannidis criteria, the evidence for preventing incisional hernias with prophylactic meshes does not meet the Class I requirements due to high heterogeneity and the presence of publication bias. However, the magnitude of the effect, the stability of the sensitivity analysis results, and the large number of patients (25,286) suggest a level of evidence approaching *Class II–III (high to suggestive*).

#### Abdominal Wound Dehiscence (AWD)

**FIGURE 3:**
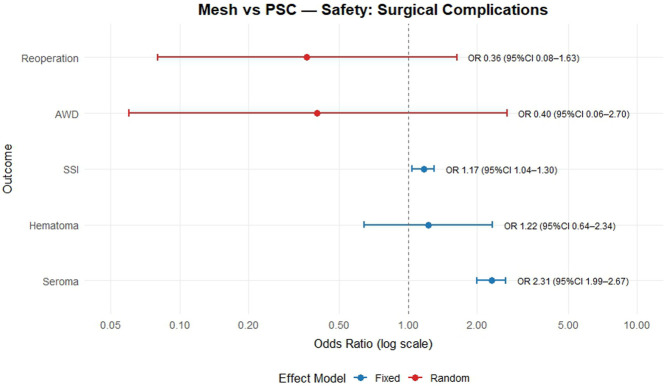
Mesh vs. PSC. Safety = surgical complications.

Only 5 meta-analyses discussed the prevention of abdominal wound dehiscence (23.8%) none of which identified a significant difference between the mesh and primary closure (OR = 0.4; 95% CI: 0.06–2.7) (Figure 3).

### Safety: SSI, Seroma, Hematoma and Reoperation

In the safety analysis (which included four main outcomes), sixteen studies (76%) reported on surgical site infection (SSI) with moderate heterogeneity (I^2^ = 37.0%; p < 0.06), and the corresponding fixed-effects model yielded an OR of 1.17 (95% CI: 1.04–1.30). Seroma was evaluated in fourteen studies (71.4%) exhibiting similar heterogeneity (I^2^ = 36.9%; p < 0.08), and the fixed-effects model yielded an OR of 2.31 (95% CI: 1.99–2.67). Three studies (14.2%) that evaluated hematoma did not demonstrate evidence of heterogeneity (I^2^ = 0%; p = 0.69), and the OR was 1.22 (95% CI: 0.64–2.34) according to a fixed-effects model. Finally, five studies (23.8%) reported on reoperation with considerable heterogeneity (I^2^ = 61.3%; p < 0.03), and a random-effects model yielded an OR of 0.36 (95% CI: 0.08–1.63) (Figure 3).

#### Other Outcomes

Because no meta-analysis specifically reported on the type of mesh, placement space, or other important variables such as cost, quality of life, or postoperative pain (although several primary studies did), this analysis was not performed.

## Discussion

This umbrella meta-analysis, considered the highest level of methodological synthesis, evaluated the efficacy (in terms of preventing IH and AWD) and safety (in terms of the occurrences of surgical site infection, hematoma and seroma) of the use of prophylactic meshes. Our findings indicate that the use of meshes significantly reduces the incidence of IH (OR = 0.29; RRR ≈71%); this effect was consistent across multiple surgical contexts, including elective, emergency, AAA and bariatric procedures.

Despite identifying high heterogeneity and evidence of possible publication bias, we consider these results to be results for three reasons: first, the stability of the effect size in the sensitivity analysis (OR = 0.30–0.42), second, an interval of consistently protective prediction (0.22–0.37) that anticipates favorable results in future studies, and third, a moderate study overlap (CCA = 0.18) that indicates a diverse but complementary evidence base.

These results demonstrate a consistent association between prophylactic mesh use and a reduced incidence of IH, although its impact on AWD could not be conclusively established. The global evidence was classified as Class II–III (high or suggestive) according to the Ioannidis criteria, thus reflecting methodological limitations inherent in the available literature; however, the magnitude of the effect, its cross-sectional consistency and the consolidated sample size (n = 25,286) support a selective role for prophylactic meshes in clinical practice, thereby suggesting active surveillance of possible biases in future publications.

**FIGURE 4:**
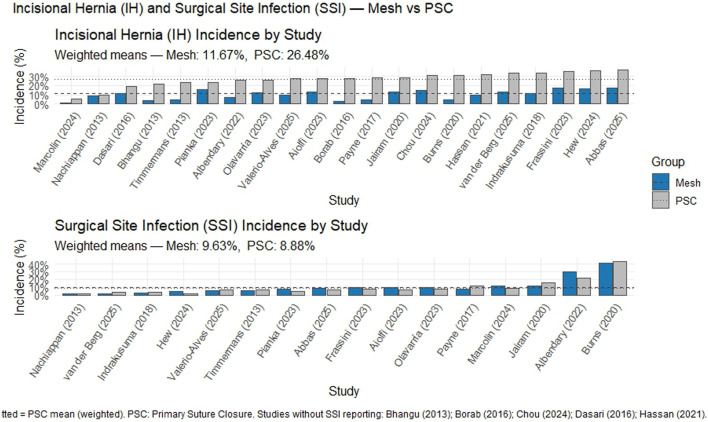
IH and SSI. Mesh vs. PSC.

Another fundamental aspect of this analysis is related to the safety of mesh placement. In our study, the use of prophylactic meshes was associated with a statistically significant increase in the risk of surgical site infection (OR≈1.17) and seroma (OR≈2.31) (Figure 4). Previous studies have analyzed this relationship by reporting the number needed to treat for net effect (NNTnet), which quantitatively integrates efficacy (including the prevention of incisional hernia) and safety (including SSO) in a single metric. In the study by Lozada et al. [8], the NNT for benefits (NNTB) was 7.57, thus indicating that approximately 8 patients should be treated with a mesh to prevent one incisional hernia, whereas the number needed to elicit harm (NNTH) was −14.3, indicating that for every 14 patients treated, one patient would experience an additional adverse event attributable to the use of the mesh. Critically, the resulting NNTnet was 5, which indicates that for every 5 patients treated with a prophylactic mesh, a net benefit would be obtained from one event (including the prevention of an incisional hernia that exceeds the occurrence of a complication). These findings support the notion that despite the increased risk of complications, the benefit-risk balance may be favorable in carefully selected high-risk patients, thereby reinforcing a context-dependent safety and efficacy profile rather than a universal preventive strategy.

An additional aspect to consider is the economic feasibility of mesh prophylaxis. Although this meta-analysis could not directly evaluate this factor because of the lack of reporting of economic data in the included primary studies, a cost-benefit analysis performed by Sheikh et al. regarding stoma closure [36] revealed that the profitability of this intervention critically depends on the type of utilized mesh. This study revealed that the use of biological meshes substantially increases costs, whereas the implementation of synthetic meshes significantly reduces costs, thereby maintaining the clinical benefits of prevention. These findings suggest that the selection of prosthetic material is a determining factor for the economic sustainability of this preventive strategy.

Although our meta-analysis could not directly assess the effect of mesh position because of the limited information reported in the primary meta-analyses, the current evidence suggests that the onlay and retromuscular planes are the most effective positions for preventing IH. The network meta-analyses of Aiolfi et al. and Tansawet et al. [37, 38] revealed that these positions exhibit the lowest probability of hernia without increasing complications such as seroma, hematoma or infection. The onlay position is particularly advantageous in contexts with limited experience in retrorectal dissection because of its lower technical complexity. In addition, this position offers the possibility of an easier and safer removal of the mesh, if necessary, thus preserving the other anatomical spaces for an eventual correction of incisional hernia in the future.

Regarding the relationship between the effectiveness of the mesh in preventing IH and its association with an increased risk of SSI, the question arises as to what other measures associated with the use of meshes could be beneficial. Although the meta-analysis by McGeehan et al. [39] did not demonstrate a significant reduction in SSI with the implementation of bundles in emergency surgery, when considering our findings, we propose that future research should evaluate the incorporation of the mesh within a specific and multimodal bundle to determine whether this strategy can reduce the risk of infection without compromising its effectiveness in preventing hernias.

Our study revealed that the use of prophylactic meshes increases the risk of infections (SSIs) and seromas. On this basis, the risks and benefits of this technique should be carefully considered. As described by Krpata et al. [40, 41], the preventive use of mesh leads to an ethical dilemma, wherein all of the treated patients are exposed to the risks of implantation with foreign material (with a complication rate close to 5.6%) to avoid hernias that may only form in some patients. This dilemma is even more relevant given safter and effective alternatives to mesh use, such as the small bites closure technique [42] or the reinforced tension line (RTL) technique [43], which reduce the formation of hernias without increasing the risk of infection. Therefore, the focus in future studies should not be simply “whether a mesh should be used” but to correctly identify those patients exhibiting such a high risk of hernia that the benefits of mesh use clearly outweigh its proven risks.

Among the limitations of the study, high heterogeneity was observed, which was associated with the use of different surgical techniques, mesh types and populations. A risk of publication bias was also identified, which could lead to an overestimation of the actual benefits. Moreover, the analysis was unable to assess crucial outcomes such as chronic pain, quality of life or costs, which limits the overall assessment of the benefit-risk balance. Finally, the significant increase in local complications such as infections (SSIs) and seromas represents a critical clinical disadvantage that must be weighed against the reduction in hernias.

## Conclusion

The use of prophylactic mesh is associated with a significant reduction in the incidence of incisional hernia (OR = 0.29), consistent relative effect across surgical contexts), supporting a preventive role rather than confirming definitive efficacy. This benefit must be interpreted alongside an increase in local complications such as seroma formation and surgical site infection, which warrants equal consideration and careful risk–benefit assessment at the individual patient level.

The global evidence demonstrated a moderate-to-suggestive level of certainty (Class II–III, according to the Ioannidis criteria), which was supported by a broad evidence base; however, considerable heterogeneity and signs of publication bias were observed. Accordingly, prophylactic mesh should not be regarded as a universal preventive strategy, and future efforts should focus on refined risk stratification of high-risk patients, evaluation of safer technical approaches, and studies addressing cost-effectiveness and quality-of-life outcomes to better define its role in incisional hernia prevention.

## References

[1] Indrakusuma R Jalalzadeh H van der Meij JE Balm R Koelemay MJW . Prophylactic Mesh Reinforcement Versus Sutured Closure to Prevent Incisional Hernias After Open Abdominal Aortic Aneurysm Repair via Midline Laparotomy: A Systematic Review and Meta-Analysis. Eur J Vasc Endovasc Surg (2018) 56(1):120–8. 10.1016/j.ejvs.2018.03.021 29685678

[2] Dasari M Wessel CB Hamad GG . Prophylactic Mesh Placement for Prevention of Incisional Hernia After Open Bariatric Surgery: A Systematic Review and Meta-Analysis. Am J Surg (2016) 212(4):615–22.e1. 10.1016/j.amjsurg.2016.06.004 27659158

[3] Chou JT Nickel I Bugaev N Hojman HM Johnson B Kim WC Prophylactic Nonabsorbable Mesh Augmentation Reduces the Risk of Incisional Ventral Hernia Following Midline Laparotomy. Curr Probl Surg (2024) 61(11):101590. 10.1016/j.cpsurg.2024.101590 39477677

[4] Rastegarpour A Cheung M Vardhan M Ibrahim MM Butler CE Levinson H . Surgical Mesh for Ventral Incisional Hernia Repairs: Understanding Mesh Design. Plast Surg (Oakv) 2016 Spring (2016) 24(1):41–50. 10.4172/plastic-surgery.1000955 27054138 PMC4806756

[5] Jairam AP López-Cano M Garcia-Alamino JM Pereira JA Timmermans L Jeekel J Prevention of Incisional Hernia After Midline Laparotomy with Prophylactic Mesh Reinforcement: A Meta-Analysis and Trial Sequential Analysis. BJS Open (2020) 4(3):357–68. 10.1002/bjs5.50261 32057193 PMC7260413

[6] Basta MN Kozak GM Broach RB Messa CA Rhemtulla I DeMatteo RP Can we Predict Incisional Hernia? Development of a Surgery-Specific Decision-Support Interface. Ann Surg (2019) 270(3):544–53. 10.1097/SLA.0000000000003472 31318790

[7] Fischer JP Harris HW López-Cano M Hope WW . Hernia Prevention: Practice Patterns and Surgeons' Attitudes About Abdominal Wall Closure and the Use of Prophylactic Mesh. Hernia (2019) 23(2):329–34. 10.1007/s10029-019-01894-z 30734888

[8] Lozada Hernández EE Maldonado Barrios IL Amador Ramírez S Rodríguez Casillas JL Hinojosa Ugarte D Smolinski Kurek RL Surgical Site Occurrence After Prophylactic Use of Mesh for Prevention of Incisional Hernia in Midline Laparotomy: Systematic Review and Meta-Analysis of Randomized Clinical Trials. Surg Endosc (2024) 38(2):942–56. 10.1007/s00464-023-10509-9 37932603

[9] Deerenberg EB Henriksen NA Antoniou GA Antoniou SA Bramer WM Fischer JP Updated Guideline for Closure of Abdominal Wall Incisions from the European and American Hernia Societies. Br J Surg (2022) 26:znac302–1250. 10.1093/bjs/znac302 36026550 PMC10364727

[10] Muysoms FE Antoniou SA Bury K Campanelli G Conze J Cuccurullo D European Hernia Society Guidelines on the Closure of Abdominal Wall Incisions. Hernia (2015) 19(1):1–24. 10.1007/s10029-014-1342-5 25618025

[11] Abdullah SH Ahmad MH . Enhancing Clarity and Methodological Rigor in Umbrella Reviews. Ann Med Surg (Lond) (2024) 86(10):6352–4. 10.1097/MS9.0000000000002536 39359834 PMC11444639

[12] Pamporis K Bougioukas KI Karakasis P Papageorgiou D Zarifis I Haidich AB . Overviews of Reviews in the Cardiovascular Field Underreported Critical Methodological and Transparency Characteristics: A Methodological Study Based on the Preferred Reporting Items for Overviews of Reviews (PRIOR) Statement. J Clin Epidemiol (2023) 159:139–50. 10.1016/j.jclinepi.2023.05.018 37245702

[13] Page MJ McKenzie JE Bossuyt PM Boutron I Hoffmann TC Mulrow CD The PRISMA 2020 Statement: An Updated Guideline for Reporting Systematic Reviews. BMJ (2021) 372:n71. 10.1136/bmj.n71 33782057 PMC8005924

[14] Lozada E Martin-del-Campo LA , Luis Alberto Fernandez Vazquez Mellado. Efficacy and Safety of Prophylactic Mesh Reinforcement for Prevention of Incisional Hernia: An Umbrella Review of Meta-Analyses. PROSPERO 2025 CRD420251125560 (2025). Available online at: https://www.crd.york.ac.uk/PROSPERO/view/CRD420251125560 (Accessed December 12, 2025). 10.3389/jaws.2026.15631 PMC12968044 41810389

[15] Shea BJ Reeves BC Wells G Thuku M Hamel C Moran J AMSTAR 2: A Critical Appraisal Tool for Systematic Reviews that Include Randomised or Non-Randomised Studies of Healthcare Interventions, or both. BMJ (2017) 358:j4008. 10.1136/bmj.j4008 28935701 PMC5833365

[16] Hennessy EA Johnson BT . Examining Overlap of Included Studies in Meta-Reviews: Guidance for Using the Corrected Covered Area Index. Res Synth Methods (2020) 11(1):134–45. 10.1002/jrsm.1390 31823513 PMC8555740

[17] Ioannidis JP . Integration of Evidence from Multiple Meta-Analyses: A Primer on Umbrella Reviews, Treatment Networks and Multiple Treatments Meta-Analyses. CMAJ (2009) 181(8):488–93. 10.1503/cmaj.081086 19654195 PMC2761440

[18] Abbas AW Abo-Elsoad MF Hindawi MD Zeid MA Kalmoush AE Aboelkier MM Prophylactic Mesh Reinforcement in Elective Abdominal Surgeries: A Systematic Review, Meta-Analysis, and GRADE Evidence Assessment. Hernia (2025) 29(1):230. 10.1007/s10029-025-03421-9 40646274 PMC12254175

[19] Aiolfi A Cavalli M Gambero F Mini E Lombardo F Gordini L Prophylactic Mesh Reinforcement for Midline Incisional Hernia Prevention: Systematic Review and Updated Meta-Analysis of Randomized Controlled Trials. Hernia (2023) 27(2):213–24. 10.1007/s10029-022-02660-4 35920944

[20] Olavarria OA Dhanani NH Bernardi K Holihan JL Bell CS Ko TC Prophylactic Mesh Reinforcement for Prevention of Midline Incisional Hernias: A Publication Bias Adjusted Meta-Analysis. Ann Surg (2023) 277(1):e162–e169. 10.1097/SLA.0000000000004729 33630465

[21] Albendary M Mohamedahmed AYY Alamin A Rout S George A Zaman S . Efficacy and Safety of Mesh Closure in Preventing Wound Failure Following Emergency Laparotomy: A Systematic Review and Meta-Analysis. Langenbecks Arch Surg (2022) 407(4):1333–44. 10.1007/s00423-021-02421-4 35020082

[22] Hew CY Rais T Antoniou SA Deerenberg EB Antoniou GA . Prophylactic Mesh Reinforcement Versus Primary Suture for Abdominal Wall Closure After Elective Abdominal Aortic Aneurysm Repair with Midline Laparotomy Incision: Updated Systematic Review Including Time-To-Event Meta-Analysis and Trial Sequential Analysis of Randomized Controlled Trials. Ann Vasc Surg (2024) 109:149–61. 10.1016/j.avsg.2024.06.026 39025216

[23] Bhangu A Fitzgerald JE Singh P Battersby N Marriott P Pinkney T . Systematic Review and Meta-Analysis of Prophylactic Mesh Placement for Prevention of Incisional Hernia Following Midline Laparotomy. Hernia (2013) 17(4):445–55. 10.1007/s10029-013-1119-2 23712289

[24] Pianka F Werba A Klotz R Schuh F Kalkum E Probst P The Effect of Prophylactic Mesh Implantation on the Development of Incisional Hernias in Patients with Elevated BMI: A Systematic Review and Meta-Analysis. Hernia (2023) 27(2):225–34. 10.1007/s10029-022-02675-x 36103010 PMC10126020

[25] Borab ZM Shakir S Lanni MA Tecce MG MacDonald J Hope WW Does Prophylactic Mesh Placement in Elective, Midline Laparotomy Reduce the Incidence of Incisional Hernia? A Systematic Review and Meta-Analysis. Surgery (2017) 161(4):1149–63. 10.1016/j.surg.2016.09.036 28040255

[26] Burns FA Heywood EG Challand CP Lee MJ . Is there a Role for Prophylactic Mesh in Abdominal Wall Closure After Emergency Laparotomy? A Systematic Review and Meta-Analysis. Hernia (2020) 24(3):441–7. 10.1007/s10029-019-02060-1 31641872 PMC7210219

[27] Frassini S Calabretto F Granieri S Fugazzola P Massaro M Sargenti B Prophylactic Mesh Augmentation After Laparotomy for Elective and Emergency Surgery: Meta-Analysis. BJS Open (2023) 7(4):zrad060. 10.1093/bjsopen/zrad060 37504969 PMC10375944

[28] Nachiappan S Markar S Karthikesalingam A Ziprin P Faiz O . Prophylactic Mesh Placement in High-Risk Patients Undergoing Elective Laparotomy: A Systematic Review. World J Surg (2013) 37(8):1861–71. 10.1007/s00268-013-2046-1 23584462

[29] Payne R Aldwinckle J Ward S . Meta-Analysis of Randomised Trials Comparing the Use of Prophylactic Mesh to Standard Midline Closure in the Reduction of Incisional Herniae. Hernia (2017) 21(6):843–53. 10.1007/s10029-017-1653-4 28864937

[30] Hassan MA Yunus RM Khan S Memon MA . Prophylactic Onlay Mesh Repair (POMR) Versus Primary Suture Repair (PSR) for Prevention of Incisional Hernia (IH) After Abdominal Wall Surgery: A Systematic Review and Meta-Analysis. World J Surg (2021) 45(10):3080–91. 10.1007/s00268-021-06238-6 34279690

[31] Timmermans L de Goede B Eker HH van Kempen BJ Jeekel J Lange JF . Meta-Analysis of Primary Mesh Augmentation as Prophylactic Measure to Prevent Incisional Hernia. Dig Surg (2013) 30(4-6):401–9. 10.1159/000355956 24217341

[32] Marcolin P Mazzola Poli de Figueiredo S Oliveira Trindade B Bueno Motter S Brandão GR Mao RD Prophylactic Mesh Augmentation in Emergency Laparotomy Closure: A Meta-Analysis of Randomized Controlled Trials with Trial Sequential Analysis. Hernia (2024) 28(3):677–90. 10.1007/s10029-023-02943-4 38252397

[33] Valério-Alves AP Saggin CLDS de Aguiar Portela JME Viana P Guerra GB de Paiva Reis CM Prophylactic Mesh Versus Primary Closure in Emergency and Elective Surgeries: A Systematic Review and Meta-Analysis of Randomized Clinical Trials. Hernia (2024) 29(1):14. 10.1007/s10029-024-03202-w 39549074

[34] van den Berg R Den Hartog FPJ Baart SJ Bali C Matsagkas M Bevis PM European Hernia Society Prophylactic Mesh Study Group Collaborators. A Systematic Review and Independent Patient Data Meta-Analysis of Prophylactic Mesh Augmentation for Incisional Hernia Prevention After Abdominal Aortic Aneurysm Surgery (I-PREVENT-AAA) A Collaborative European Hernia Society Project. Ann Surg (2025) 26. 10.1097/SLA.0000000000006684 PMC12695395 40008430

[35] Pieper D Antoine SL Mathes T Neugebauer EA Eikermann M . Systematic Review Finds Overlapping Reviews Were Not Mentioned in Every Other Overview. J Clin Epidemiol (2014) 67(4):368–75. 10.1016/j.jclinepi.2013.11.007 24581293

[36] Sheikh Y Asunramu H Low H Gakhar D Muthukumar K Yassin H A Cost-Utility Analysis of Mesh Prophylaxis in the Prevention of Incisional Hernias Following Stoma Closure Surgery. Int J Environ Res Public Health (2022) 19(20):13553. 10.3390/ijerph192013553 36294132 PMC9602752

[37] Aiolfi A Bona D Gambero F Sozzi A Bonitta G Rausa E What Is the Ideal Mesh Location for Incisional Hernia Prevention During Elective Laparotomy? A Network Meta-Analysis of Randomized Trials. Int J Surg (2023) 109(5):1373–81. 10.1097/JS9.0000000000000250 37026844 PMC10389496

[38] Tansawet A Numthavaj P Techapongsatorn S Wilasrusmee C Attia J Thakkinstian A . Mesh Position for Hernia Prophylaxis After Midline Laparotomy: A Systematic Review and Network Meta-Analysis of Randomized Clinical Trials. Int J Surg (2020) 83:144–51. 10.1016/j.ijsu.2020.08.059 32927135

[39] McGeehan G Edelduok IM Bucholc M Watson A Bodnar Z Johnston A Systematic Review and Meta-Analysis of Wound Bundles in Emergency Midline Laparotomy Identifies that It Is Time for Improvement. Life (Basel) (2021) 11(2):138. 10.3390/life11020138 33670186 PMC7916918

[40] Krpata D . Evaluating Lay Perception of Prophylactic Mesh Placement: There Are Risks, Benefits, and Alternatives. J Surg Res (2019) 237:87–8. 10.1016/j.jss.2017.11.064 29273430

[41] Weissler JM Carney MJ Enriquez FA Messa CA Broach R Shapira MM Using Crowdsourcing as a Platform to Evaluate Lay Perception of Prophylactic Mesh Placement. J Surg Res (2019) 237:78–86. 10.1016/j.jss.2017.11.065 29290370

[42] Deerenberg EB Harlaar JJ Steyerberg EW Lont HE van Doorn HC Heisterkamp J Small Bites Versus Large Bites for Closure of Abdominal Midline Incisions (STITCH): A Double-Blind, Multicentre, Randomised Controlled Trial. Lancet (2015) 386(10000):1254–60. 10.1016/S0140-6736(15)60459-7 26188742

[43] Lozada Hernández EE Flores González E Chavarría Chavira JL Hernandez Herrera B Rojas Benítez CG García Bravo LM The MESH-RTL Project for Prevention of Abdominal Wound Dehiscence (AWD) in High-Risk Patients: Noninferiority, Randomized Controlled Trial. Surg Endosc (2024) 38(12):7634–46. 10.1007/s00464-024-11358-w 39453454

