# Supplementary material for: Efficacy and Safety of Prophylactic Mesh Reinforcement for the Prevention of Incisional Hernia: An Umbrella Review of Meta-Analyses
Source: J Abdom Wall Surg. 2026 Feb 23;5:15631. doi: 10.3389/jaws.2026.15631 (PMC12968044; doi:10.3389/jaws.2026.15631)

**Table A.** Search strategy

| **Online databases** | **Search strategy** |
| --- | --- |
| **PubMed** | ("Incisional Hernia"[tiab] OR "Abdominal Wall Hernia"[tiab]) AND ("Mesh"[tiab] OR "Prophylactic Mesh"[tiab] OR "Mesh Reinforcement"[tiab]) AND ("Laparotomy"[Mesh] OR "Abdominal Surgery"[tiab] OR "Midline Laparotomy"[tiab]) AND ("Systematic Review"[Publication Type] OR "Meta-Analysis"[Publication Type] OR "systematic review"[tiab] OR "meta-analysis"[tiab]) |
| **The Cochrane Library** | ("Incisional Hernia" OR "Abdominal Wall Hernia") AND (Mesh OR "Prophylactic Mesh" OR "Mesh Reinforcement") AND ("Laparotomy" OR "Abdominal Surgery" OR "Midline Laparotomy") AND ("systematic review" OR "meta-analysis") |
| **SCOPUS** | TITLE-ABS-KEY(("Incisional Hernia" OR "Abdominal Wall Hernia") AND (Mesh OR "Prophylactic Mesh" OR "Mesh Reinforcement") AND ("Laparotomy" OR "Abdominal Surgery" OR "Midline Laparotomy") AND ("systematic review" OR "meta-analysis")) |
| **ScienceDirect** | ("Incisional Hernia" OR "Abdominal Wall Hernia") AND (Mesh OR "Prophylactic Mesh" OR "Mesh Reinforcement") AND ("Laparotomy" OR "Abdominal Surgery" OR "Midline Laparotomy") AND ("systematic review" OR "meta-analysis") |
| **Google Scholar** | Incisional Hernia "Abdominal Wall Hernia" Mesh "Prophylactic Mesh" "Mesh Reinforcement" Laparotomy "Midline Laparotomy" "systematic review" OR "meta-analysis" |

| **Table B.** Updated AMSTAR 2 Evaluation | | | | | | | |  |
| --- | --- | --- | --- | --- | --- | --- | --- | --- |
| **AMSTAR 2 Criteria** | **Aiolfi et al. (2023)** | **Olavarría et al. (2023)** | **Albendary et al. (2022)** | **Jairam et al. (2020)** | **Hew et al. (2024)** | **Bhangu et al. (2013)** | **Pianka et al. (2023)** |  |
| **1. Protocol registered before the review?** | ✓ | ✗ | ✗ | ✓ | ✓ | ✗ | ✓ |  |
| **2. Rationale for the meta-analysis clearly justified?** | ✓ | ✓ | ✓ | ✓ | ✓ | ✓ | ✓ |  |
| **3. Comprehensive literature search performed?** | ✓ | ✓ | ✓ | ✓ | ✓ | ✓ | ✓ |  |
| **4. Were at least two independent reviewers involved in study selection?** | ✓ | ✓ | ✓ | ✓ | ✓ | ✓ | ✓ |  |
| **5. Were at least two independent reviewers involved in data extraction?** | ✓ | ✓ | ✓ | ✓ | ✓ | ✓ | ✓ |  |
| **6. Was the risk of bias of included studies assessed using an appropriate tool?** | ✓ | ✓ | ✓ | ✓ | ✓ | ✗ | ✓ |  |
| **7. Were the study designs appropriate for the research question?** | ✓ | ✓ | ✓ | ✓ | ✓ | ✓ | ✓ |  |
| **8. Were appropriate statistical methods used to combine results?** | ✓ | ✓ | ✓ | ✓ | ✓ | ✓ | ✓ |  |
| **9. Was the risk of bias considered when interpreting results?** | ✓ | ✓ | ✓ | ✓ | ✓ | ✓ | ✓ |  |
| **10. Was heterogeneity assessed using appropriate methods?** | ✓ | ✓ | ✓ | ✓ | ✓ | ✓ | ✓ |  |
| **11. Was publication bias considered and assessed?** | ✓ | ✗ | ✓ | ✓ | ✓ | ✗ | ✓ |  |
| **12. Were subgroup analyses or sensitivity analyses conducted appropriately?** | ✓ | ✓ | ✓ | ✓ | ✓ | ✗ | ✓ |  |
| **13. Were sources of funding and conflicts of interest of included studies considered?** | ✓ | ✓ | ✓ | ✓ | ✓ | ✗ | ✓ |  |
| **14. Was the funding of the meta-analysis reported?** | ✓ | ✓ | ✓ | ✓ | ✓ | ✓ | ✓ |  |
| **15. Was the overall quality of the evidence assessed? (e.g., GRADE)** | ✓ | ✓ | ✓ | ✓ | ✓ | ✗ | ✓ |  |
| **16. Was the study conducted according to high methodological standards?** | ✓ | ✓ | ✓ | ✓ | ✓ | △ | ✓ |  |
| **Final AMSTAR 2 Rating** | **HIGH Quality** | **MODERATE to HIGH Quality** | **HIGH Quality** | **HIGH Quality** | **HIGH Quality** | **MODERATE Quality** | **HIGH Quality** |  |
| ✓ = Criterion met \| ✗ = Criterion not met \| △ = Partial compliance / Some concerns | | | | | | | |  |
|  |  |  |  |  |  |  |  |  |
|  |  |  |  |  |  |  |  |  |
|  |  |  |  |  |  |  |  |  |
| **AMSTAR 2 Criteria** | **Borab et al. (2017)** | **Burns et al. (2020)** | **Chou et al. (2024)** | **Dasari et al. (2016)** | **Frassini et al. (2023)** | **Indrakusuma et al. (2018)** | **Nachiappan et al. (2013)** |  |
| **1. Protocol registered before the review?** | ✓ | ✓ | ✓ | ✓ | ✓ | ✓ | ✗ |  |
| **2. Rationale for the meta-analysis clearly justified?** | ✓ | ✓ | ✓ | ✓ | ✓ | ✓ | ✓ |  |
| **3. Comprehensive literature search performed?** | ✓ | ✓ | ✓ | ✓ | ✓ | ✓ | ✓ |  |
| **4. Were at least two independent reviewers involved in study selection?** | ✓ | ✓ | ✓ | ✓ | ✓ | ✓ | ✓ |  |
| **5. Were at least two independent reviewers involved in data extraction?** | ✓ | ✓ | ✓ | ✓ | ✓ | ✓ | ✓ |  |
| **6. Was the risk of bias of included studies assessed using an appropriate tool?** | ✓ | ✓ | ✓ | ✓ | ✓ | ✓ | ✓ |  |
| **7. Were the study designs appropriate for the research question?** | ✓ | ✓ | ✓ | ✓ | ✓ | ✓ | ✓ |  |
| **8. Were appropriate statistical methods used to combine results?** | ✓ | ✓ | ✓ | ✓ | ✓ | ✓ | ✓ |  |
| **9. Was the risk of bias considered when interpreting results?** | ✓ | ✓ | ✓ | ✓ | ✓ | ✓ | ✓ |  |
| **10. Was heterogeneity assessed using appropriate methods?** | ✓ | ✓ | ✓ | ✓ | ✓ | ✓ | ✓ |  |
| **11. Was publication bias considered and assessed?** | ✓ | ✗ | ✓ | ✓ | ✓ | ✓ | ✗ |  |
| **12. Were subgroup analyses or sensitivity analyses conducted appropriately?** | ✓ | ✗ | ✓ | ✓ | ✓ | ✓ | ✗ |  |
| **13. Were sources of funding and conflicts of interest of included studies considered?** | ✓ | ✗ | ✓ | ✓ | ✓ | ✓ | ✗ |  |
| **14. Was the funding of the meta-analysis reported?** | ✓ | ✓ | ✓ | ✓ | ✓ | ✓ | ✓ |  |
| **15. Was the overall quality of the evidence assessed? (e.g., GRADE)** | ✓ | ✓ | ✓ | ✓ | ✓ | ✓ | ✓ |  |
| **16. Was the study conducted according to high methodological standards?** | ✓ | ✓ | ✓ | ✓ | ✓ | ✓ | ✓ |  |
| **Final AMSTAR 2 Rating** | **HIGH Quality** | **MODERATE to HIGH Quality** | **HIGH Quality** | **HIGH Quality** | **HIGH Quality** | **HIGH Quality** | **MODERATE to HIGH Quality** |  |
|  |  |  |  |  |  |  |  |  |
|  |  |  |  |  |  |  |  |  |
| **AMSTAR 2 Criteria** | **Hassan et al. (2021)** | **Timmermans et al. (2013)** | **Marcolin et al. (2024)** | **Valerio-Alves et al. (2025)** | **Abbas et al. (2025)** | **Payne et al. (2017)** | **van der Berg et al. (2025)** |  |
| **1. Protocol registered before the review?** | ✓ | ✗ | ✓ | ✓ | ✗ | ✗ | ✗ |  |
| **2. Rationale for the meta-analysis clearly justified?** | ✓ | ✓ | ✓ | ✓ | ✓ | ✓ | ✓ |  |
| **3. Comprehensive literature search performed?** | ✓ | ✓ | ✓ | ✓ | ✓ | ✓ | ✓ |  |
| **4. Were at least two independent reviewers involved in study selection?** | ✓ | ✓ | ✓ | ✓ | ✓ | ✓ | ✓ |  |
| **5. Were at least two independent reviewers involved in data extraction?** | ✓ | ✓ | ✓ | ✓ | ✓ | ✓ | ✓ |  |
| **6. Was the risk of bias of included studies assessed using an appropriate tool?** | ✓ | ✓ | ✓ | ✓ | ✓ | ✓ | ✓ |  |
| **7. Were the study designs appropriate for the research question?** | ✓ | ✓ | ✓ | ✓ | ✓ | ✓ | ✓ |  |
| **8. Were appropriate statistical methods used to combine results?** | ✓ | ✓ | ✓ | ✓ | ✓ | ✓ | ✓ |  |
| **9. Was the risk of bias considered when interpreting results?** | ✓ | ✓ | ✓ | ✓ | ✓ | ✓ | ✓ |  |
| **10. Was heterogeneity assessed using appropriate methods?** | ✓ | ✓ | ✓ | ✓ | ✓ | ✓ | ✓ |  |
| **11. Was publication bias considered and assessed?** | ✓ | ✓ | ✓ | ✓ | ✗ | ✗ | ✗ |  |
| **12. Were subgroup analyses or sensitivity analyses conducted appropriately?** | ✓ | ✓ | ✓ | ✓ | ✓ | ✗ | ✗ |  |
| **13. Were sources of funding and conflicts of interest of included studies considered?** | ✓ | ✓ | ✓ | ✓ | ✓ | ✗ | ✗ |  |
| **14. Was the funding of the meta-analysis reported?** | ✓ | ✓ | ✓ | ✓ | ✓ | ✓ | ✓ |  |
| **15. Was the overall quality of the evidence assessed? (e.g., GRADE)** | ✓ | ✓ | ✓ | ✓ | ✓ | ✓ | ✓ |  |
| **16. Was the study conducted according to high methodological standards?** | ✓ | ✓ | ✓ | ✓ | △ | ✓ | ✓ |  |
| **Final AMSTAR 2 Rating** | **HIGH Quality** | **HIGH Quality** | **HIGH Quality** | **HIGH Quality** | **MODERATE Quality** | **MODERATE to HIGH Quality** | **MODERATE to HIGH Quality** |  |

**Figure A.** Funnel plot. Umbrella Meta analysis for IH.


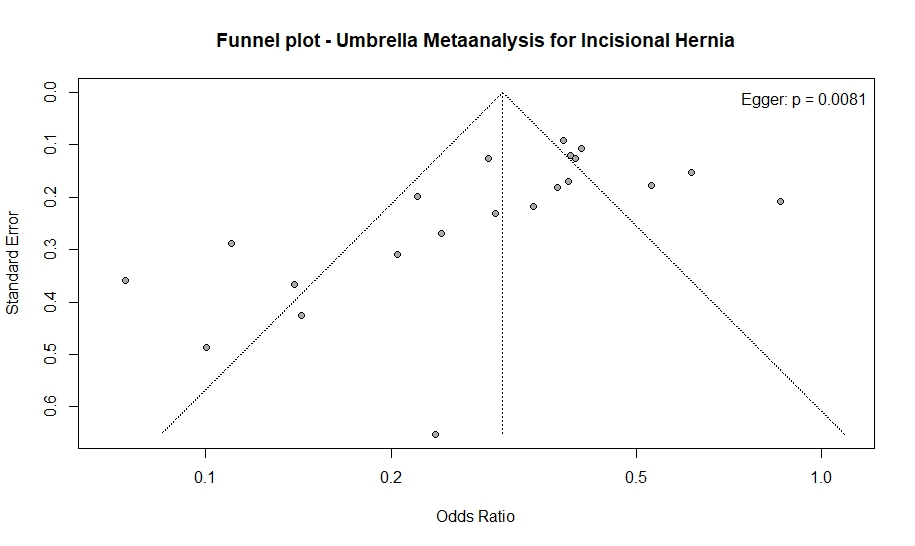


**Figure B.** Leave-One-Out sensitivity analysis


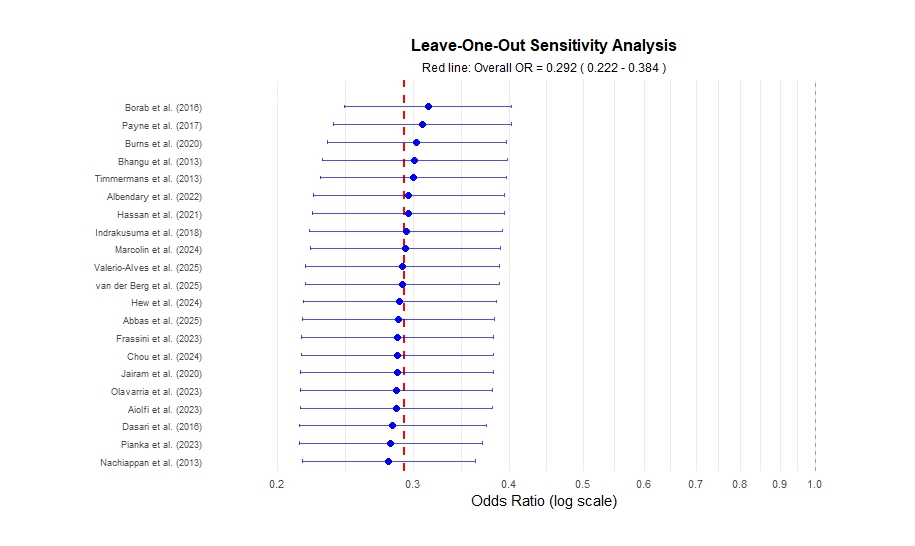

Supplement: Supplementary file 1 [file Supplementaryfile1.docx]
